# La singularité du patient tuberculeux dans le système de santé: l'expérience du Burkina Faso

**DOI:** 10.11604/pamj.2013.15.22.2103

**Published:** 2013-05-13

**Authors:** Roger Zerbo

**Affiliations:** 1 Institut des sciences des sociétés (INSS-CNRST) / membre du Laboratoire de recherche interdisciplinaire en sciences de la santé Université de Ouagadougou (LARISS)-Burkina Faso

**Keywords:** Patient tuberculeux, santé publique, anthropologie, système de santé, TB patient, public health, anthropology, Health System

## Abstract

La démarche pluridisciplinaire s'impose de plus en plus dans le secteur de la santé dans le contexte africain, où les parcours thérapeutiques sont pluriels et les rapports des usagers des centres de santé sont complexes et peu satisfaisants. La compréhension des représentations de la maladie est nécessaire pour offrir des soins appropriés certes, mais l'expérience individuelle des malades représente également une source de savoir. Les données ont été collectées à travers des entretiens semi-directifs, le recueil des récits de vie et des observations participantes. Cet article rend compte d'une expérience d'implication de socio-anthropologue dans la mise en œuvre d'un programme de santé publique au Burkina Faso en vue d'une amélioration de la prise en charge des malades tuberculeux. L'analyse des données à été conduite dans une visée réflexive. Les perspectives socio-anthropologiques ont révélé que l'expérience des anciens malades tuberculeux peut être mise à profit afin d'apporter des changements dans les relations thérapeutiques et l'intégration sociale des autres malades. Cette idée à été mise en application pour tenir lieu d'une traduction des analyses anthropologiques en actes pour un changement. L'article évoque la manière dont l'approche socio-anthropologique au sein d'un programme de santé, peut mettre en évidence le potentiel des malades à être des acteurs importants dans le fonctionnement des services de soins et leur propre bien-être. Dans cette situation, la démarche théorique implique la réflexivité de l'anthropologue, mais également un regard critique sur les modes d'intervention en santé publique.

## Introduction

Diverses formes d'applications des résultats provenant d’études anthropologiques sont de plus en plus constatées dans des programmes de santé et de développement dans plusieurs milieux et ce, depuis les années 1960 [1, 2]. Elles répondent à une vision d'amélioration des conditions de vie et de santé des malades, une bonne organisation des soins de santé. Cette perspective de l'anthropologie soulève des questions d'ordre méthodologiques et épistémologiques dans le développement des recherches en sciences sociales [3]. Par ailleurs nous pouvons avancer que les éléments d'application de l'anthropologie sont consubstantiels à la pratique anthropologique, dans la mesure où ses conclusions ne manquent d'influencer les décideurs de manière directe ou indirecte [4]. De nos jours, les sollicitations des anthropologues dans la réalisation de projets sont récurrentes. Au cours des trois dernières décennies, leur implication dans des programmes de santé, notamment ceux autour du Sida et de la tuberculose, a contribué à l'amélioration des performances des politiques et des centres de soins [5], mais aussi a permis un apport substantiel au développement de l'anthropologie fondamentale [6]. L'implication des anthropologues dans les programmes de santé ne caractérise-t-elle pas une nouvelle manière de conduire des recherches sur les dynamiques sociales ‘ De nombreux auteurs ont envisagé cette perspective avec des expériences multiformes dans divers contextes [7]. C'est assurément une opportunité de recherches anthropologiques et en ce sens, Laurent Vidal [8] invite à faire des systèmes de santé, « une fabrique de l'anthropologie ». Nous avons pu prendre la mesure de cette approche à travers notre expérience d'implication dans de nombreux programmes de santé réalisés au Burkina Faso durant une période allant de 2003 à 2010. Ces programmes de santé s'intéressaient aux questions relatives à la tuberculose, le VIH/Sida et le paludisme. Dans le contexte Ouest-africain, ces contributions mettent en évidence les dysfonctionnements des systèmes de santé, les difficiles interactions entre malades et soignants, mais aussi la désillusion des prestataires de soins vis-à-vis de leur condition de vie et de travail [9]. Ces contributions faisant état de l'implication des chercheurs en sciences sociales dans les activités du secteur de la santé, visent la promotion de la santé en mettant en perspective les concepts qui émanent de l'Organisation Mondiale de la Santé auxquels les politiques et les agents de santé des pays s'ajustent. Cette approche est d'autant plus pertinente quand il s'agit des maladies chroniques parmi lesquelles on peut citer la tuberculose qui a des implications d'ordre social et psychologique [10]. Cet article retrace et analyse les modalités d'implication des anthropologues dans la réalisation d'un programme de santé, le projet « Formation Recherche-Action en Santé » (FORESA) caractérisé par une démarche de recherche-action participative au Burkina Faso. Le propos consiste ici à montrer les stratégies développées et les enjeux relatifs à une telle approche pour la recherche en sciences sociales et la prise en compte des besoins des malades. La pertinence de cette approche relève du fait qu'il est de plus en plus recommandé de développer une approche globale des problèmes de santé des malades tuberculeux [11]. Un intérêt particulier est accordé à l'analyse théorique dans le but de mettre en évidence le caractère urgent de la problématique. La liberté d'expression des anthropologues dans le cadre d'un projet de santé, ainsi que les difficultés de conduire leurs travaux dans le cadre d'un projet de santé et de développement ne sont pas ignorées.

La tuberculose est un problème de santé publique, notamment à cause du Sida et elle pose le problème d'une anthropologie des systèmes de santé [12]. Elle entraine des problèmes sociaux et économiques importants dans le vécu quotidien des personnes touchées; notamment leur état de pauvreté s'accentue à cause de la maladie, ainsi que l'a constaté Paul Farmer [13]. Elle évoque également des problèmes en rapport avec le fonctionnement des centres de santé [14]. L'anthropologie s'intéressant aux représentations sociales de la maladie reconnaît qu'elle pose également des questions liées au fonctionnement des institutions sociales et aux interactions sociales entre individus [15–16]. Au Burkina Faso, les proportions de malades dont le traitement ne donne pas les résultats escomptés sont d'environ 10,2% en 2006 et les taux de succès au traitement varient de 60,2% à 68,6 en fonction des localités du Burkina Faso [17]. Outre les recommandations de l'organisation mondiale de la santé concernant la décentralisation des soins vers les centres de santé en zones rurales périphériques, l'intérêt de considérer le malade dans sa globalité est de plus en plus démontré [18]. Dans le cas du Burkina Faso, la stratégie adoptée est basée sur le traitement sous observation directe (TOD) du malade tuberculeux durant toute la durée de son traitement qui varie de six à huit mois au moins. Dans une approche anthropologique, l'analyse des relations thérapeutiques est pertinente en soi, ainsi que l'ont fait Eliot Freidson [19] dans le contexte Américain dans les années 1970 Jean-Pierre Olivier de Sardan & Yannick Jaffré [20] dans le contexte Africain. Ceci appréhende la dynamique des relations thérapeutiques et la qualité des soins, qui s'avère être une analyse importante pour éclairer les décideurs politiques, les intervenants et les administrateurs des services de santé. Néanmois, inclure ces données dans le cadre d'une recherche-action reste un défi pour les acteurs des systèmes de santé [21]. Depuis 2001, dans le cadre des projets de santé publique, les politiques sanitaires envisagent de prendre en compte les propositions des acteurs communautaires, des professionnels de la santé et des malades pour améliorer l'offre de soins aux malades. Toutefois, les résultats sont toujours insatisfaisants. Certaines valeurs et principes inhérents à l'approche des sciences sociales expriment l'intérêt de ces disciplines pour l'amélioration du fonctionnement des services de santé. Au tour des systèmes de soins, la tuberculose a des implications sociales et relationnelles qui s'expriment à travers le comportement des malades et des soignants. Nous nous inscrivons dans la logique d'une problématique récurrente qui est de savoir comment engager l'anthropologie dans le développement et le changement social ‘ Les contributions lors du Colloque international de l'association euro-africaine pour l'anthropologie du changement social et du développement (APAD) tenu en janvier 2010 à Ouagadougou, ont planché sur cette problématique en présentant des expériences multiples. Il reste bien entendu que le débat autour des applications de l'anthropologie est toujours d'actualité [7], mais ce n'est pas le propos de cet article. Il s'agit de rendre compte de l'implication de l'approche anthropologique dans une action de santé publique dans le cadre d'un système complexe que représente le centre de santé, dans une perspective de prise en compte des réalités sociales locales. La réflexion porte sur les possibilités de réhabiliter la singularité du malade impliqué dans un projet de recherche/action.

## Description du contexte du projet et les enjeux de sante publique

Le contexte qui a permis de récolter des données empiriques qui alimentent cet article est matérialisé par un dispositif impliquant des chercheurs en santé publiques, des anthropologues, des économistes de la santé, des administrateurs de services de santé, et des prestataires de soins. Il s'agissait pour l'anthropologue de conduire des recherches et d’élaborer des connaissances qui sont applicables et utilisables immédiatement par les autres acteurs des systèmes de santé. Et l'argument d'impliquer un malade dans le fonctionnement des services de santé a été convaincant. Non seulement l'expérience est documenté, mais la nécessité d'avoir une attitude réflexive s'impose comme une posture indispensable à l’élaboration d'une connaissance anthropologique. Un anthropologue qui réalise des travaux sur commande, ne devrait pas perdre de vue la nécessité de prendre du recul par rapport à son engagement, et ce à quoi il a abouti. Ce qui explique le nombre important de références bibliographiques en faveur de la réflexivité en regard d'une démarche d'anthropologie impliquée. Dans notre cas, il s'agit notamment d'expérimenter et de partager l'expérience de réflexivité à laquelle tout anthropologue devrait faire face dans ses travaux.

Comme objectifs de départ, il s'est agit principalement de recherches anthropologiques dans l'accompagnement des interventions de santé publique, dans la direction régionale sanitaire du plateau central (DRPCL) de 2005 à 2009, au Burkina Faso. Mais, notons que les constats vont au-delà de cette période et de cette expérience. La DRPCL est l'une des 13 régions sanitaires que compte le Burkina Faso. La région se compose de trois districts sanitaires que sont les districts de Boussé, de Ziniaré et de Zorgho. Chaque district dispose d'un centre médical avec antenne chirurgicale (CMA) fonctionnel. Elle couvre 3 provinces, celle de Oubritenga, de Ganzourgou et du Kourwéogo. Le chef lieu de la région administrative est Ziniaré, ville située à environ 35 kilomètres au Nord de Ouagadougou, la capitale du Burkina Faso. La superficie totale de la région est de 8 453 km2. Pour l'année 2007, la population était estimée à 760 148 habitants. Selon le cadre stratégique régional de lutte contre la pauvreté, 58.6% de la population vit en dessous du seuil de pauvreté. Plusieurs programmes de santé publique sont mis en œuvre dont le projet FORESA. Ce programme qui a permis d'avoir des subsides pour la recherche et la mise en œuvre des activités est financée par l'Union Européenne, dans le cadre d'une collaboration entre le programme national tuberculose (PNT-Burkina Faso), l'Institut de Recherche en Sciences de la Santé (IRSS-Burkina Faso) et l'Ecole de santé Publique de l'Université Libre de Bruxelles (ESP/ULB) en Belgique.

Outre la collaboration institutionnelle entre des centres de recherches universitaires (ULB, IRSS) et des centres de soins (PNT, DRPCL), le programme de santé est mis en œuvre selon une démarche pluridisciplinaire impliquant des anthropologues, des économistes de la santé, des chercheurs en sciences de la santé et des prestataires de soins. Sa réalisation est caractérisée sur des échanges d'expériences, une approche réflexive et principalement la prise en compte des besoins des malades dans leurs milieux. Le rôle des anthropologues, consistait à mettre en œuvre des études qualitatives à but exploratoire et d'indiquer des modalités d'application des résultats dans une perspective de recherche-action et de recherche-évaluative. De manière générale, il est connu que dans le cadre de l'exécution des programmes de santé qui combinent un volet recherche à un volet d'intervention, c'est qu'il a été convenu de faire face à une transversalité des problématiques de recherches, qui recommande plusieurs approches et convoquant des compétences professionnelles différentes. Ainsi se matérialise-t-elle la promotion des approches pluridisciplinaires. On reconnaît également que les acteurs cibles des interventions ont des compétences et sont en mesure de participer collectivement à la résolution des problèmes de développement par une mise en évidence de leurs compétences. La mise en œuvre du projet FORESA a été une opportunité offerte aux chercheurs, gestionnaires des centres de soins, et agents de santé, de s'approprier une méthode de travail en collaboration et une stratégie d'action qui les rend aptes à la réflexivité et leur permet d'agir avec clairvoyance. Ce dispositif d'approche pluridisciplinaire crée chez les acteurs impliqués une sensibilité vis-à-vis des disciplines qui développent des approches parfois différentes, tout en valorisant la spécificité et la complémentarité des disciplines. Cette disposition a permis de tenir compte de l'approche anthropologique qui révèle la singularité du tuberculeux. Ainsi, au fil des analyses anthropologiques portant sur le mode de fonctionnement des systèmes de santé, axées sur les modalités de prise en charge de la tuberculose, nous avons progressivement accumulé des éléments qui retracent les itinéraires des malades en rapport avec les prestations de soins, et les différents mécanismes de recomposition des relations sociales. Ceci révèle les facteurs de dysfonctionnements des services de santé ainsi que les rapports complexes du malade à son environnement.

## Procédés de sélection et rôles du patient-expert dans le système de soin

La stratégie consiste à prendre attache avec les malades qui, de manière volontaire donnent leur accord, néanmoins, moyennant quelques indemnisations, à l'amélioration de la prise en charge des nouveaux malades. Cette indemnisation représente l'achat d'une bicyclette à 115 euros environ, et le payement de 2,5 euros à l'occasion des rencontres trimestrielles de mise au point.

### Les critères de choix des malades étaient les suivants

Avoir été atteint de la tuberculose, quelle que soit la forme, Avoir suivi le traitement jusqu’à la guérison attestée par un examen médical de confirmation Décider volontairement, à l'issue d'un entretien libre avec un soignant, ensuite avec un anthropologue ou un psychologue, à partager son expérience avec les autres malades Accepter de contribuer de manière bénévole à l'animation d'un club de malades tuberculeux et être impliqué dans les programmes d’éducation sanitaire

### Les activités proposées au patient sont les suivantes

Expliquer, à la demande de l'agent de santé, les exigences du protocole de soins antituberculeux, en langage simple et compréhensible au malade parfois analphabète. Cela implique que celui-ci doit pouvoir s'exprimer facilement dans la langue courante de la localité; animer un club ou un groupe d'observance thérapeutique, constitué de malades sous traitement; faire des témoignages en cas de besoin, des possibilités de guérison pour rassurer les malades; aider à l'observance en anticipant sur les effets indésirables liés aux médicaments antituberculeux; participer à la recherche des absents au traitement, ainsi qu’à la distribution des vivres aux malades indigents.

Au-delà de ces critères, le recueil des récits de vie des malades, dans le cadre de la recherche anthropologique permettait d'identifier des anciens malades potentiels dont le parcours pourrait permettre d'encourager certains nouveaux malades pour leur prise en charge. A cet effet, douze récits composés de témoignages directs rapportés par des accompagnants de malades, des prestataires de soins et des expériences vécues par les malades ont été récoltés. Les langues de dialogue et de communication utilisées étaient soit le « Mooré », le « Djula », ou le Français.

Un “corpus” de données constitué à la fois à partir des entretiens individuels semi-directifs avec le personnel de santé, les nouveaux et anciens malades et leurs proches, les membres d'association a servi à l'analyse des conditions de vie des tuberculeux, en lien avec les représentations et la gestion sociale de la maladie, ainsi que la perception des pratiques de soins. Des discussions collectives ont été organisées sous formes d’échanges d'expériences. Quinze récits de vie de malades tuberculeux et cinq expériences de pratiques professionnelles d'agents de santé ont été récoltés et analysés. Des observations de la pratique des agents de santé et les visites aux domiciles des malades ont aussi permis de comprendre le comportement des acteurs et leur rapport à la tuberculose. Outre ces observations directes comme « attitude cognitive » qui donnent accès au vécu individuel des personnes auxquelles on s'intéresse. Les constats de terrain ont été confrontés aux expériences rapportées dans la littérature dans d'autres contextes plus ou moins similaires en guise de comparaison. A titre d'illustration, un ancien malade de la tuberculose, nommé Eloi, qui a fait preuve d'un dynamisme et d'une grande motivation, est impliqué dans des actions communautaires, rend visite régulièrement aux nouveaux malades tuberculeux afin de les aider à gérer ou à préserver leurs processus d'intégration sociale. Ces visites sont généralement effectuées dans les centres de santé, mais également dans les domiciles. Au cours d'un échange, il confie les propos ci-après:

*J'ai été désigné comme responsable de l'association des anciens malades de la tuberculose dans le district sanitaire de Ziniaré. Je ne peux passer trois jours sans rentrer au dispensaire. Lorsque j'arrive, je fais le tour des malades, je leur adresse mes salutations, puis je leur demande s'ils ont pris leurs médicaments. Je suis arrivé au centre de santé un jour et j'ai vu un homme qui était assis, c’était un tuberculeux. Je l'ai salué et il m'a répondu. J'ai demandé ce qu'il y a; il a dit que ça va. Il suit le traitement mais il n'a personne qui l'assiste. Je l'ai demandé depuis combien de temps il est ici, et il m'a répondu que ça vaut deux semaines. Il m'a dit alors que si j'habite dans la localité, de l'aider. Je lui ai demandé de quel quartier il vient; il a dit qu'il vient du village de bsouya, situé à 35 Km de la ville de Ziniaré. J'ai demandé s'il connaît le chef de ce village. Il a dit qu'ils n'ont pas de lien particulier. Je suis allé voir le chef de village de bsouya. Il m'a conseillé d'aller au commissariat voir un gars et nous sommes revenus voir le malade. On lui a posé quelques questions. Le monsieur du commissariat est retourné voir le chef de village de bsouya. Dans l'après-midi du même jour, des gens de la famille du malade sont venus le rendre visite. (...). Donc ce jour-là, le malade était tellement content, et le lendemain, je suis revenu le voir (...). Il m'a dit que sa maladie était déjà guérie parce qu'il est content, et que même s'il meurt maintenant, il sera dans la paix. C'est ainsi et il a retrouvé ses proches. Il a son ami qui a appris ce que j'ai fait, et il est venu me remercier (...) »*.

Cet ancien malade de la tuberculose, après sa guérison apportait son soutien à des nombreux malades qui en avaient besoin et communiquait certaines inquiétudes manifestées par des malades auprès des agents de santé. Son engagement et la richesse de son expérience étaient particuliers. Sa bonne volonté dans le processus d'amélioration des conditions de prise en charge des malades lui a permis de résoudre plusieurs difficultés liés à la stigmatisation des malades en regard des perceptions parfois erronées des étiologies de la tuberculose.

## Discussion

### Problématiques de recherche-action et éléments d'analyses théoriques

Deux problématiques sont développées dans cet article. D'une part, l'implication des anthropologues dans la mise en œuvre du programme de santé, d'autre part la prise en compte de la singularité du patient-expert dans la réalisation des programmes de santé. Il s'agit de démontrer par la documentation d'une expérience concrète de recherche pluridisciplinaire, que l'un des rôles de l'anthropologue, et plus largement l'apport de l'anthropologie aux actions de santé publique, c'est d’être attentif à la dynamique des interactions entre les acteurs des systèmes de santé. Cette sensibilité vis-à-vis la personnalité des acteurs, permet d'envisager une nouvelle stratégie de collaboration pour améliorer leurs interactions. L'anthropologue a eu pour tâche de faire l’état des lieux du fonctionnement des mécanismes de prise en charge des malades tuberculeux et de proposer une nouvelle manière de fonctionner. C'est ainsi que la prise en compte du malade tuberculeux comme étant un patient-expert a été expérimenté avec succès.

Il s'agit d'une démarche dont il est convenu d'appeler anthropologie impliquée, très largement adoptée par des anthropologues travaillant sur la problématique du Sida et plus généralement dans le secteur de la santé publique [6]. L'anthropologue est parfois appelé à appréhender son objet de l'intérieur [2]., tout comme il y a des exemples où l'on s'interroge sur le rôle des chercheurs en sciences sociales dans le déroulement des politiques de santé [22]. Les données auxquelles nous nous référons pour la rédaction de cet article proviennent d’éléments empiriques d'un processus d'implication de l'anthropologue dans une action de transformation, celle de l'amélioration des soins de santé aux malades tuberculeux.

Il s'est agi de nous poser la question de savoir, comment se façonne le « patient expert » et que peut-il apporter à la compréhension et au fonctionnement des systèmes médicaux complexes ‘ Est considéré ici comme patient expert, le malade tuberculeux qui a connu un parcours thérapeutique complexe et diversifié, une vie sociale perturbée, des relations sociales recomposées, une observance scrupuleuse des prescriptions médicales, une guérison parfaite et une prédisposition à partager son expérience. La possibilité d'associer l'individu ayant été confronté à une pathologie, dans le fonctionnement des institutions de soins a été éprouvée pour le cas des maladies chroniques tels que le diabète [23] et particulièrement le cas du VIH/Sida pour lequel les personnes séropositives sont très actives dans la définition des politiques de soins et la défense du droit des malades [24]. Cela implique qu'il a un bon niveau d'estime de soi malgré l’épisode de la maladie. Avoir un bon niveau d'estime de soi pour un ancien malade tuberculeux implique qu'il accepte son statut et évoque sans gêne les difficultés rencontrées, les stratégies adoptées pour les surmonter, et dispose effectivement de connaissances qui lui permettent de conseiller les autres malades. L'expérience met en évidence, aussi bien des objectifs de compréhension, des attitudes qui influencent les modes d'accès aux soins, mais aussi une intervention auprès des acteurs de la santé en vue d'une transformation. Au tour des systèmes de soins, la tuberculose a des implications sociales et relationnelles qui s'expriment à travers le comportement des malades et des soignants [10, 13, 28]. Il reste bien entendu que le débat autour des applications de l'anthropologie est toujours d'actualité [25]. Cette analyse réflexive est déposée dans une autre contribution et approfondie dans le cadre de ma thèse de doctorat. Le malade chronique peut être aussi bien un objet d’étude de la biomédecine que de l'anthropologie ou la psychologie [26]. C'est dans ce sens que Laurent Vidal invite à une réflexion sur l'exercice anthropologique autour du singulier [14] pour mettre en perspective l'itinéraire des malades et en faire un mode d'accès à la dynamique sociale qui entoure la maladie. Cette approche est « à mi-chemin d'une sociologie qui appréhende les faits sociaux dans leur globalité et d'une psychologie de l'individu, cette anthropologie du singulier postule que les stratégies et représentations sociales ne peuvent se décrire qu’à partir d'un regard sur leurs expressions singulières » [14]. Dans la même vision, Jean-Pierre Olivier de Sardan [27] propose une approche anthropologique qui révèle les jeux d'acteurs et l'importance d'appréhender leurs stratégies d'actions dans une visée de compréhension et de délivrance des services publiques en Afrique. Ces deux postures théoriques indiquent que la dynamique sociale qui influence les recours aux soins peut être appréhendée à travers les malades et leurs rapports aux services de soins.

### Malade tuberculeux, acteur de changement des pratiques thérapeutiques

Au Burkina Faso, les représentations sociales de la tuberculose poussent à recourir aux tradithérapeutes [28, 29]. C'est une maladie qui touche principalement les populations démunies et les personnes infectées par le VIH [17]. A travers les itinéraires thérapeutiques du tuberculeux, on constate une reconstitution de ses réseaux de relations sociales, des interactions troublées avec ses proches et les agents de santé et un processus de stigmatisation sociale qui influence sa qualité de vie. Au travers d'une reconstitution et une mise en perspective analytique de ses itinéraires de soins, le malade tuberculeux guérit se révèle être un acteur du système de santé qui peut aider à améliorer les relations thérapeutiques dans lors des soins de santé. Chaque attitude du malade obéit à une rationalité: recours tardif au centre de santé, recours aux médecines dites traditionnelles, observance d'un traitement médical contraignant, et la volonté de contribuer au fonctionnement du système de soins. Les malades qui ont traversé ces étapes ont été rencontrés et invité à partager leurs expériences avec les nouveaux tuberculeux aux côtés des agents de santé. Ceci a permis aux agents de santé de prendre conscience des manquements dans leurs pratiques, et aux nouveaux malades, de comprendre facilement le traitement. Les malades tuberculeux sont persévérants dans la recherche de soins efficaces, et les représentations sociales influencent leurs comportements. La douleur engendrée par la tuberculose pousse les gens à rechercher des soins avec parfois l'aide des proches. La tuberculose est considérée comme étant une sanction sociales dans la perception des populations, ou la conséquence d'une transgression d'interdis sociaux, par exemple le viol ou les relations sexuelles extraconjugales. Dans ce cas, la démarche de soins consiste à rétablir les liens sociaux du malade [30]. Le malade multiplie les options de recours thérapeutiques afin de recouvrer la santé, mais le retard du diagnostic occasionne des dépenses en nature comme en espèce, qui accentuent sa précarité. Dès lors que le diagnostic de la maladie est établi, le traitement biomédical aboutit dans la plupart des cas, à une guérison. En 2003 le taux de guérison était estimé par le PNT à environ 77% [17]. Des échecs de traitement et des cas de résistances aux antituberculeux sont également envisageables et même de plus en plus fréquents [17]. Les raisons du non recours spontané aux centres de soins, et la complexité du traitement de la tuberculose sont appréhendées à partir de l'expérience des malades, pour autant qu'on s'intéresse à leur itinéraire thérapeutique. La tuberculose confronte les malades à plusieurs difficultés dans ses relations sociales qu'ils gèrent plus ou moins bien en fonction de leur niveau d'estime de soi, de l’évolution de la maladie, et surtout de l'appui de l'entourage. A cause de la tuberculose, les malades sont victimes d'actes de marginalisation sociale et de stigmatisation, autant dans les ménages que dans les lieux publics. Cette attitude fait suite à la crainte de la maladie, mais aussi, trouve ses origines dans le comportement des agents de santé par qui le malade intériorise fortement sa marginalité [10]. Au regard des représentations sociales liées à la maladie, et le diagnostic populaire qui évoque l'idée d'une maladie provoquée par un tiers, les malades ont souvent recourt à la médecine traditionnelle pour entre autres, réparer un acte de transgression d'interdit culturel, ou rétablir les liens sociaux du malade [16]. Par ailleurs, le traitement de la tuberculose est confronté à des épisodes d'inobservances thérapeutiques de la part de certains malades, ce qui occasionne des cas d’échecs qui augmentent d'année en année, et un taux de succès au traitement inférieur à 80% [17]. Le fonctionnement du système de santé et les modes de prise en charge de la tuberculose devraient être interrogés afin de prendre en compte les attentes des malades et des autres acteurs afin de réduire les inégalités d'accès aux soins [31]. Ceci a permis d'appréhender les expériences des tuberculeux dans leurs interactions avec l'entourage, les modalités de gestion de la tuberculose et les pratiques de soins. La prise en compte et l'analyse des récits de vie, et des témoignages rapportés sur la vie des malades permet de comprendre les parcours thérapeutiques, et interpelle sur ce qu'on peut faire des anciens malades, si on les considère comme acteur pour l'offre de soins antituberculeux et la mise en œuvre des actions de lutte contre la tuberculose. Dans cette expérience mise en œuvre au Burkina Faso, deux anciens malades tuberculeux arrivés au terme de leur traitement qui a abouti à leur guérison complète, ont été associés à la prise en charge des malades tuberculeux. Leur rôle consistait à s'entretenir avec les nouveaux malades afin de leur expliquer davantage les exigences du traitement et à rechercher les malades qui sont irréguliers dans les centres de santé. Leur expérience et leur dynamisme ont été enrichissant pour les soignants et les nouveaux malades [21]. Cependant, l'implication des anciens malades dans le fonctionnement du système de soins, a été temporaire en raison du contexte du projet limité à 3 ans, et de l'absence d'une définition claire de leur statut dans l'organisation d'ensemble des services de soins. Il y a eu une grande influence de la logique de projet qui a mis l'accent sur une approche quasi-expérimentale de courte durée. Les aspects tels que, la communication entre soignant et soigné, l'aide à l'observance, l'approvisionnement en médicaments, ont fait l'objet d'une analyse critique, afin de dégager des solutions alternatives bénéfiques pour le bien-être des malades. Cela montre que « l'anthropologie impliquée » peut aider à résoudre des problèmes médicaux et sociaux autour de la tuberculose et des malades chroniques. En cela, l'invitation à la réflexivité des chercheurs est constante. D'après les analyses de Jean Macq & al. [32], l'augmentation du pouvoir de décision ou alors les capacités de choix des malades tuberculeux, procède de plusieurs manières: « L'expérience a montré que les définitions opérationnelles, les potentielles barrières envers l'autonomisation et les aspects contextuels doivent être prise en compte. Quatre types d'approches ont été identifiés: (a) rendre capable les malades à être plus responsables de leur santé, concernant particulièrement l'adhésion au traitement, (b) organiser les malades tuberculeux en groupes et associations, (c) élaborer des processus de soins centrés sur le malade pour la prise en charge de la tuberculose et des prestations de soins en général, (d) utiliser enfin les aptitudes en plaidoyer des malades tuberculeux pour améliorer la lutte contre la tuberculose » (traduction libre). Les principales difficultés rencontrées dans le processus d'autonomisation des tuberculeux sont entre autres les difficultés d'accès aux soins, la stigmatisation et les contraintes liées à la prise de médicament sous surveillance. Dans une approche interdisciplinaire, ce n'est pas toujours évident que les visions de l'intervention selon l'anthropologue soient pleinement adoptées par les acteurs-décideurs qui détiennent les moyens [31]. Parfois, les analyses de l'anthropologue se révèlent trop complexes et parfois abstraites. Dans certaines situations, les acteurs d'intervention n'y prêtent aucune attention selon Marc-Eric Gruénais [33]. En effet, la promotion d'une approche globale de la santé nécessite la création d'un espace de dialogue qui favorise une compréhension mutuelle des attentes des malades et des prestataires de soins dans l'organisation des services de santé [20–33]. Ceci est d'autant plus nécessaire pour le cas de la tuberculose, le Sida et les maladies chroniques en général.

## Conclusion

Le fil conducteur de l'article consiste à partir d’éléments théoriques développés par des auteurs chevronnés sur la singularité et le rôle de l'acteur dans un système complexe (le système de santé) et mettre en rapport avec le cas du malade tuberculeux, à travers une expérience concrète de réalisation d'un programme de santé. De plus, des expériences comparables dans le cas des personnes vivant avec le VIH ont également été évoquées. L'argumentaire a été bâtit autour du témoignage d'un seul malade qui reflète de nombreuses autres exemples comparables. En plus, l'expérience d'implication de l'anthropologue dans la réalisation des projets représente sans doute en elle-même une leçon pour les approches en anthropologie du développement. L'intérêt d'une démarche d'anthropologie impliquée est de favoriser les changements de perspectives des actions des politiques publiques. Cette démarche conduit à comprendre la singularité du malade tuberculeux, et faire évoluer son image habituelle de personne marginale et nécessiteuse de soins pour justifier les interventions en santé publique. Bien plus, il devient un acteur social, producteur de discours, détenteur d'expériences et de compétences, donc sources de connaissances sur les pratiques de soins par rapport auxquelles il construit son rapport au traitement et à la santé. Son engagement auprès des malades et agents de santé traduit l'importance de la dimension sociale de la maladie, les enjeux et les stratégies liés à la communication dans le processus thérapeutique de la tuberculose. On comprend comment l'objet de recherche et cible des programmes de santé à savoir le malade, est également acteur d'intervention, parce que producteur de connaissances sur les pratiques des agents de santé et capable de partager ses expériences à des fins de changement. Cette démarche a été éprouvée pour le cas de certains malades du VIH/Sida et d'autres maladies chroniques. Les malades tuberculeux, au regard de son itinéraire thérapeutique, se construit une identité et prend conscience de son rôle possible dans l'amélioration des processus de traitement et de prise en charge globale des maladies. A ce jour, les modalités de son implication ne sont quasiment pas définies dans le fonctionnement des systèmes de santé.
